# Biopsychosocial Illness Model: From the Lung to the Eye

**DOI:** 10.3390/jcm11154298

**Published:** 2022-07-25

**Authors:** Fabio Scarinci, Mariacristina Parravano, Francesca Romana Patacchioli

**Affiliations:** IRCCS—Fondazione Bietti, Via Livenza, 3, 00198 Rome, Italy; fabioscarinci@gmail.com (F.S.); mcparravano@gmail.com (M.P.)

Studies on the impact of different pathologies on the quality of life have made the translation of multidisciplinary scientific knowledge into a unified model of biopsychosocial disease possible in which several important biological variables are integrated with psychological-functional and sociological variables. From a pathogenetic point of view, many of these variables manifest themselves in apparently independent pathologies, such as those affecting the lung or the eye. This approach can contribute significantly to improving patients’ well-being by providing the tools necessary to deal with critical health situations and also through therapeutic integration with non-pharmacological approaches.

The Special Issue of the *Journal of Clinical Medicine* titled “Obstructive Sleep Apnea Syndrome: Pathogenesis, Treatments and Comorbidity” aims to outline studies targeting patients with Obstructive Sleep Apnea (OSA) using a multidisciplinary approach. The Special Issue has achieved the goal of providing useful elements for improving the quality of life of patients, highlighting how the multidisciplinary approach can contribute to improving knowledge on the pathogenetic mechanisms of OSA and related comorbidities. In fact, OSA can be truly considered a multifactorial disease as it involves the activity of researchers from different medical specialties, among which clinical psychology and ophthalmology stand out.

In particular, a recent review of the literature indicates that there is a cross-link between OSA and central serous chorioretinopathy (CSC). Both diseases share some pathogenetic elements that can be classified as biopsychosocial that contemplate the bidirectional interaction of biological challenges with psychological-functional and sociological characteristics [1].

The OSA syndrome causing a frequently severe sleep disorder includes also transient hypoxia, hypercapnia and the occurrence of excessive daytime sleepiness. Last but not least, the pathogenic role of OSA has gained increasing interest in multiple conditions, such as cardiovascular, cerebrovascular and even ocular diseases. In this regard, it has been suggested that a disorder of the activity of the hypothalamic–pituitary–adrenal (HPA) axis associated with the high perception of distress present in both patients with OSA and in patients with CSC could represent the shared path that explains their common component linked to distress [1,2]. Several studies have shown significant pathogenetic disturbances of the HPA axis activity, consisting in the flattening of the awake cortisol response (CAR) in the OSA group, while an increase in the amplitude of the CAR peak was found in patients with CSC [1,3,4]. These HPA axis anomalies could provide the functional basis for explaining the shared pathogenetic role of distress in both diseases. However, research is still needed to investigate the psycho-neuro-endocrinological aspects of OSA and CSC in order to outline the psychoeducational strategies that can be used to attenuate the negative impact of stress.

With regard to this, the traditional view of the mechanisms linking distress and disease has mainly focused on the classical stress system, represented by the HPA axis and cortisol as its main product. Moreover, the interaction between stress, sympathetic activation and the autonomic nervous system (ANS), although still under debate, may be important in the pathogenesis of OSA as well CSC.

ANS dysregulation shown by measuring salivary α-amylase (α-Amy) was highlighted in CSC patients [5,6,7]. Furthermore, it has been shown that abnormal diurnal α-Amy production was associated with OSA [3], providing pneumologists and ophthalmologists with a promising and innovative chronobiological approach for these patients [1,4,8].

Overall, we trust that the issues raised in this editorial with the ambitious goal of translating multidisciplinary scientific knowledge into unified practices could help to improve the patient’s quality of life.

## References

[1] Scarinci F., Patacchioli F.R., Ghiciuc C.M., Palmery M., Pasquali V., Bercea R.M., Cozma S., Parravano M. (2020). Psychological Profile and Distinct Salivary Cortisol Awake Response (CAR) in Two Different Study Populations with Obstructive Sleep Apnea (OSA) and Central Serous Chorioretinopathy (CSC). J. Clin. Med..

[2] Scarinci F., Ghiciuc C.M., Patacchioli F.R., Palmery M., Parravano M. (2019). Investigating the hypothesis of stress system dysregulation as a risk factor for central serous chorioretinopathy: A literature mini-review. Curr. Eye Res..

[3] Ghiciuc C.M., Dima Cozma L.C., Bercea R.M., Lupusoru C.E., Mihaescu T., Szalontay A., Gianfreda A., Patacchioli F.R. (2013). Restoring the salivary cortisol awakening response through nasal continuous positive airway pressure therapy in obstructive sleep apnea. Chronobiol. Int..

[4] Scarinci F., Patacchioli F.R., Palmery M., Pasquali V., Costanzo E., Ghiciuc C.M., Parravano M. (2019). Diurnal trajectories of salivary cortisol and α-amylase and psychological profiles in patients with central serous chorioretinopathy. Chronobiol. Int..

[5] Abdelhakim A.H., Ledesma-Gil G., Yannuzzi L.A. (2021). Salivary alpha amylase levels may correlate with central serous chorioretinopathy activity. Retina.

[6] Scarinci F., Patacchioli F.R., Costanzo E., Parravano M. (2021). Relationship of choroidal vasculature and choriocapillaris flow with alterations of salivary *α*-amylase patterns in central serous chorioretinopathy. Investig. Ophthalmol. Vis. Sci..

[7] Scholz P., Altay L., Sitnilska V., van Dijk E.H., Pereira A.M., van Haalen F.M., Fauser S., Boon C.J.F., Akhtar I. (2021). Salivary alpha amylase levels may correlate with central serous chorioretinopathy activity. Retina.

[8] Scarinci F., Patacchioli F.R., Parravano M. (2021). Exploring the Biopsychosocial Pathways Shared by Obstructive Sleep Apnea (OSA) and Central Serous Chorioretinopathy (CSC): A Literature Overview. J. Clin. Med..

